# Characterization of a Potential Probiotic *Lactobacillus brevis* RK03 and Efficient Production of γ-Aminobutyric Acid in Batch Fermentation

**DOI:** 10.3390/ijms19010143

**Published:** 2018-01-04

**Authors:** Chien-Hui Wu, Yi-Huang Hsueh, Jen-Min Kuo, Si-Jia Liu

**Affiliations:** 1 Department of Seafood Science, National Kaohsiung Marine University, Kaohsiung City 81157, Taiwan; ikuojm@webmail.nkmu.edu.tw (J.-M.K.); abc50525102@gmail.com (S.-J.L.); 2 Graduate School of Biotechnology and Bioengineering, Yuan Ze University, Taoyuan City 32003, Taiwan; yihhsueh@saturn.yzu.edu.tw

**Keywords:** gamma aminobutyric acid, monosodium glutamate, fermentation, *Lactobacillus brevis*

## Abstract

Lactic acid bacteria were isolated from fish and evaluated for their γ-aminobutyric acid (GABA)-producing abilities. Out of thirty-two isolates, *Lactobacillus brevis* RK03 showed the highest GABA production ability. The effects of various fermentation parameters including initial glutamic acid level, culture temperature, initial pH, and incubation time on GABA production were investigated via a singleparameter optimization strategy. For industrial large-scale production, a low-cost GABA producing medium (GM) broth was developed for fermentation with *L. brevis* RK03. We found that an optimized GM broth recipe of 1% glucose; 2.5% yeast extract; 2 ppm each of CaCO_3_, MnSO_4_, and Tween 80; and 10 μM pyridoxal phosphate (PLP) resulted in a maximum GABA yield of 62,523 mg/L after 88 h following the addition of 650 mM monosodium glutamate (MSG), for a conversion rate of 93.28%. Our data provide a practical approach for the highly efficient and economic production of GABA. In addition, *L. brevis* RK03 is highly resistant to gastric acid and bovine bile salt. Thus, the discovery of *Lactobacillus* strains with the ability to synthesize GABA may offer new opportunities in the design of improved health-promoting functional foods.

## 1. Introduction

γ-Aminobutyric acid (GABA) is a non-protein amino acid synthesized by glutamic acid decarboxylase (GAD), a pyridoxal-5′-phosphate-dependent enzyme. GAD catalyzes the irreversible α-decarboxylation of L-glutamic acid to GABA [1,2]. GABA constitutes a major inhibitory neurotransmitter in the sympathetic nervous system and has antidepressant [3], antihypertensive [4], and anti-diabetic effects in humans [5]. GABA can regulate blood pressure, heart rate, sensations of pain and anxiety, and serum lipid levels, as well as assist with insulin secretion to prevent diabetes [6,7]. Therefore, GABA has become a bioactive component of pharmaceuticals and foods. It is produced by various microorganisms such as fungi [8], yeasts [9], and lactic acid bacteria (LAB) [10]. In particular, LAB have garnered attention from the food industry because they are generally regarded as safe (GRAS) organisms for GABA production. Several GABA-producing LAB have been reported, including *Lactobacillus fermentum* [11], *Lactobacillus futsaii* [12], *Lactobacillus brevis* [3,13,14,15,16,17,18], *Lactobacillus delbrueckii* subsp. *bulgaricus* [19], *Lactococcus lactis* [20], *Lactobacillus paracasei* [21], *Lactobacillus plantarum* [22], and *Lactobacillus senmaizukei* [23].

Various fermentation factors affect the GABA production rate, the most commonly tested and essential being incubation time, initial pH, incubation temperature, and initial glutamic acid concentration [16]. Fermentation conditions can be optimized by assessing a single variable at a time. For example, the highest GABA production was achieved by *L. brevis* at initial pH 5.0 [15]. A similar optimal pH was found for *L. paracasei* NFRI 7415 when cells were incubated in medium supplemented with 500 mM monosodium glutamate (MSG) [21]. Additionally, in *Streptococcus salivarius* subsp. *thermophilus* Y2, fermentation condition optimization yielded a pH of 4.5 [24]. *L. brevis* GABA100, isolated from fermenting black raspberry juice, produced maximum GABA levels at pH 3.5 [25]. Therefore, the optimum conditions of fermenting microorganisms vary according to the different properties of GADs, with optimal pH ranging from pH 3.5–5.0.

Furthermore, Villegas et al. found that adding 270 mM of MSG to Man, Rogosa and Sharpe (MRS) medium produced a maximum GABA yield by *L. brevis* CRL 194 of approximately 270 mM [16]. Komatsuzaki et al. added 500 mM MSG to produce approximately 302 mM GABA using *L. paracasei* NFRI 7415 [20]. Li et al. [26] and Zhang et al. [17] used 3% and 7% MSG for fermentation, producing 149.05 mM and 38 g/L of GABA, respectively, using *L. brevis*. Therefore, the range for MSG appears to be 149.05–500 mM.

Many researchers have identified an optimal temperature of 30 °C, although Yang et al. found that for GABA fermentation to be 37 °C [21]. In terms of trace elements, Ueno et al. [27] and Seo et al. [28] showed that sulfur ions and calcium can activate GAD enzyme activity, respectively. Li et al. found that adding Tween 80 can enhance GABA production [14], as could CaCl_2_, MgSO_4_, and MnSO_4_, as demonstrated by Seo et al. [28].

In addition to their potential probiotic characteristics, several LAB strains can also produce GABA during their metabolism process; however, not all of these strains can be used as probiotics because some also produce biogenic amines [29,30], such as histamine [31], tyramine [30], and putrescine [29,30], which are toxic to the human body. Therefore, it is important to test LAB for their probiotic properties and potential for toxin production. For instance, Son et al. isolated a *L. brevis* KU15006 strain from the traditional Korean pickle, kimchi. It has antidiabetic properties and anti-adhesion activity against foodborne pathogens [32]. Aarti et al. isolated *L. brevis* LAP2 from a fermented fish food, Henta in North-East India and found that it has probiotic and antioxidant properties [33]. 

Therefore, the aim of this study was to evaluate GABA-producing LAB from saltwater fish and optimize the fermentation conditions for maximum GABA production using a single-variable optimization design and the highest-producing isolate, *L. brevis* RK03 from the red bigeye fish (*Priacanthus macracanthus)* from southern Taiwan. Furthermore, the antibiotic susceptibilities, in vitro acid and bile tolerance, and survival rate in simulated gastrointestinal (GI) juice and intestinal juice for *L. brevis* RK03 have been evaluated. However, the characteristics of probiotics for *L. brevis* RK03 still need to be fully examined in the future.

## 2. Results

### 2.1. Isolation of a High-GABA-Producing LAB from Saltwater Fish

**Figure 1:**
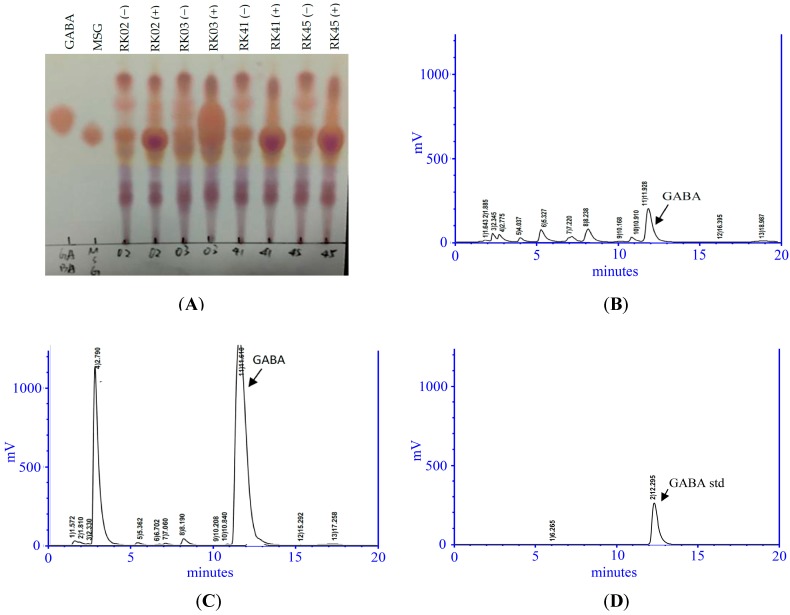
Characteristics of *L. brevis* RK03 isolated from *Priacanthus macracanthus*. (**A**) Thin layer chromatography (TLC) profiles of γ-aminobutyric acid (GABA) and monosodium glutamate (MSG) standards and selected filtration of lactic acid bacteria (LAB) cultures with (+) or without (−) MSG. High performance liquid chromatography (HPLC) analysis of supernatants from *L. brevis* RK03 cells cultured in: (**B**) Man, Rogosa and Sharpe (MRS) medium; and (**C**) MRS medium supplement with 1% MSG; (**D**) GABA standard. These data represent two different separate experiments.

We isolated thirty-two putative LAB strains from the intestines of nine fish. Strain RK03 showed the highest GABA production (1024 mg/L) as measured using HPLC (Table S1). As shown in Figure 1A, TLC analysis of a GABA standard and RK03 showed that RK03 is able to produce GABA in MRS and MRS supplemented with 1% MSG. Similar results from HPLC chromatographic analyses are shown in Figure 1B,C. The GABA standard retention time was approximately 12.291 ± 0.011 min (Figure 1D).

Strain RK03 was further characterized by 16S rRNA gene sequencing. We used two primers to amplify a fragment of approximately 510 bp, and the sequence was analyzed and aligned with genomic sequences from NCBI. RK03 exhibited 99% identity to the *L. brevis* type strain ATCC14869 and therefore was identified as *Lactobacillus brevis* RK03.

### 2.2. Effect of Initial Glutamic Acid Concentrations on Growth Profile and GABA Production

To determine whether the amount of initial glutamic acid affected GABA production, we grew bacterial cells under fixed fermentation parameters (initial pH 4.5; culture temperature 30 °C; incubation time 96 h) in MRS medium supplemented with different glutamic acid concentrations ranging from 0 to 650 mM. We observed increases in GABA production by increasing the initial glutamic acid concentration from 200 to 650 mM, with the maximum GABA yield of 15,143 mg/L obtained at 550 mM (Table 1). Cell growth was not significantly affected by modifying the initial MSG concentration. Similar results were observed by Zhang et al. [17]. 

### 2.3. Effect of Initial pH and Temperature on Growth Profile and GABA Production

We further examined the effect of cell inoculum variation from 1 × 10^7^ to 1 × 10^9^ CFU/mL on bacterial growth profile and GABA production in MRS and MRS with glutamic acid (550 mM) for 96 h (Table 1). *L. brevis* RK03 produced the highest GABA amounts at 1 × 10^9^ CFU/mL inoculum, yielding 773 and 14,443 mg/L in MRS and MRS with glutamic acid, respectively. Upon varying culture temperature from 25 °C to 45 °C, *L. brevis* RK03 produced the highest GABA amounts at a temperature of 30 °C, yielding 1230 and 21,936 mg/L in the respective media. Examination of initial pH values from pH 3.5–6.5 indicated that *L. brevis* RK03 produced the highest GABA amounts at pH 4.5, yielding 983 and 25,359 mg/L in the respective media (Table 1). Culture medium pH changes with incubation time during fermentation; therefore, the initial pH influenced the final biomass and GABA production. Overall, the best conditions for bacterial growth and GABA production were pH 4.5, 30 °C, and 1 × 10^9^ CFU/mL inoculum, with maximum GABA production observed at 550 mM MSG.

### 2.4. Effect of Organic Carbon and Nitrogen Sources on Growth Profile and GABA Production

**Figure 2:**
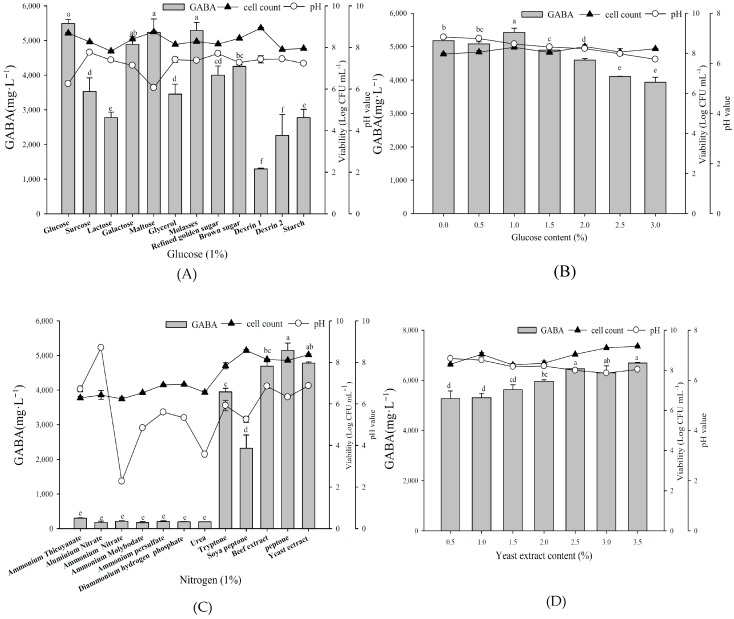
The effects of various carbon and nitrogen sources on GABA production by *L. brevis* RK03. Bacterial cells were grown with: (**A**) different carbon sources at 1%, 1% yeast extract and 1% MSG; (**B**) different percentages of glucose, 1% yeast extract, and 1% MSG; (**C**) different organic nitrogen sources at 1%, 1% glucose, and 1% MSG; and (**D**) different percentages of yeast extract, 1% glucose, and 1% MSG at 30 °C for 96 h. Different lowercase letters indicate statistically significant differences of GABA production. These data represent two different separate experiments. Data are expressed as mean ± SD from triplicate experiments. Different letters at the top of the bars are significantly different (*p* < 0.05).

To determine the optimal carbon and nitrogen sources for fermentation, *L. brevis* RK03 was first grown with different carbon sources (all at 1%), along with 1% yeast extract and 1% MSG (Figure 2A), with 1% glucose resulting in maximum GABA production. Additionally, among different glucose concentrations from 0.5% to 3.0%, 1.0% glucose again yielded maximum GABA production (Figure 2B). Among organic nitrogen sources at 1%, peptone yielded the maximum production, closely followed by 1% yeast extract (Figure 2C). However, as peptone is much more expensive than yeast extract, we selected the latter for fermentation. Maximum GABA production was obtained by 2.5% and 3.5% yeast extract among different concentrations tested (Figure 2D). In conclusion, we selected 1% glucose and 2.5% yeast extract for further fermentation.

### 2.5. Effect of Various Substances on Growth Profile and GABA Production

**Figure 3:**
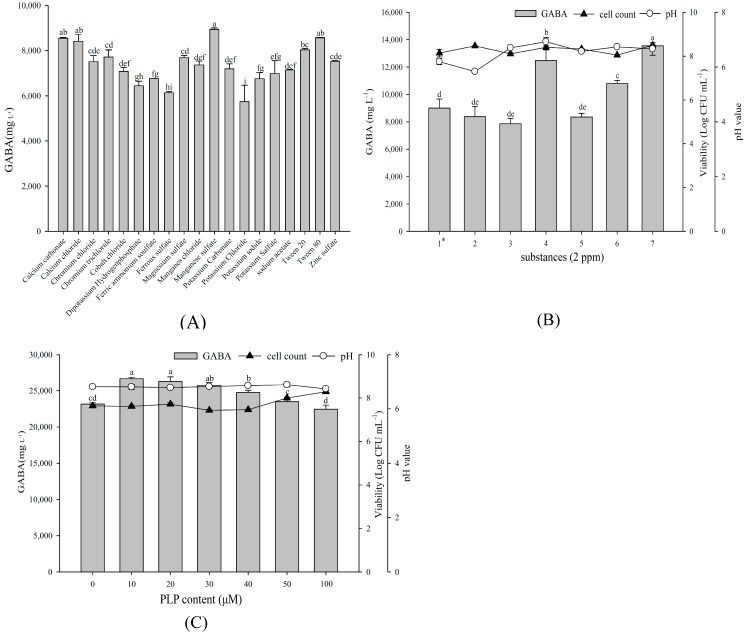
The effects of various substances on GABA production, cell viability and final pH values. (**A**) Bacterial cells were grown at 1% MSG, 1% glucose, and 2.5% yeast extract, and different substances at 2 ppm. Bacterial cells were cultivated with: (**B**) selected substances for 96 h at 30 °C; and (**C**) at different PLP contents for 96 h at 30 °C. 1 *: calcium carbonate; 2: manganese sulfate; 3: Tween 80; 4: calcium carbonate and manganese sulfate; 5: calcium carbonate and Tween 80; 6: manganese sulfate and Tween 80; 7: calcium carbonate, manganese sulfate, and Tween 80. Data are expressed as mean ± SD from triplicate experiments. Different letters at the top of the bars are significantly different (*p* < 0.05).

Taking 1% glucose, 2.5% yeast extract, and 1% MSG as a base medium, we investigated various substances to determine whether they enhance GABA production. As shown in Figure 3A, supplementing 2 ppm additional MnSO_4_ most increased the maximum GABA production, followed by CaCO_3_ and Tween 80. Subsequent growth factor of substances combination demonstrated that 2 ppm each of CaCO_3_, MnSO_4_, and Tween 80 yielded the maximum GABA production (Figure 3B). In addition, the presence of pyridoxal phosphate (PLP), a GAD cofactor, can increase GABA production. Among different PLP concentrations, *L. brevis* RK03 yielded the maximum GABA production at 10 or 20 μM PLP (Figure 3C). These findings indicate 2 ppm each of CaCO_3_, MnSO_4_, plus Tween 80 and 10 μM PLP as improving GABA production by *L. brevis* RK03.

### 2.6. Optimal MSG Concentrations in G Broth on Growth Profile and GABA Production

**Figure 4:**
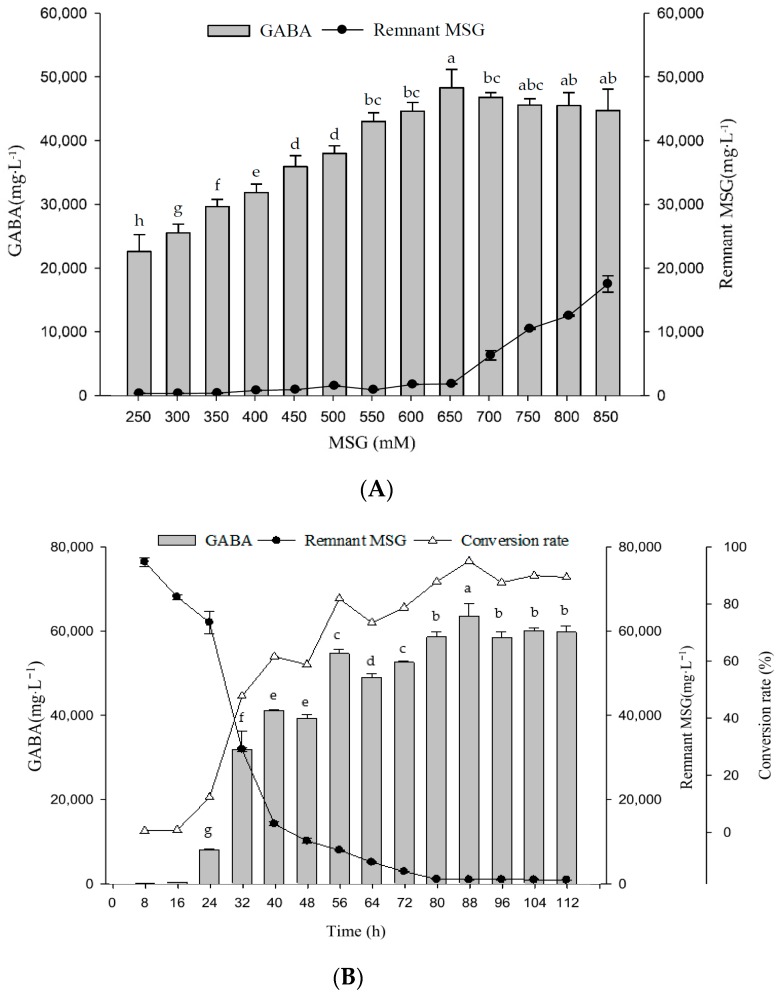
The effect of different amount of MSG in G broth on GABA production. (**A**) Bacterial cells were grown in 500 mL G broth with different concentrations of MSG for 96 h at 30 °C. G broth contains 1% glucose, 2.5% yeast extract, 2 ppm calcium carbonate, 2 ppm manganese sulfate, 2 ppm Tween 80 and 10 uM PLP. Different lowercase letters indicate statistically significant differences of GABA production. (**B**) Bacterial cells were grown in 2 L G broth with 650 mM MSG (GM broth) in 3 L flasks at 30 °C for 112 h. Data are expressed as mean ± SD from triplicate experiments. Different letters at the top of the bars are significantly different (*p* < 0.05).

For GABA production in *L. brevis* RK03, we defined the optimal medium (G broth) as containing 1% glucose; 2.5% yeast extract; 2 ppm each of CaCO_3_, MnSO_4_, and Tween 80; and 10 μM PLP. We then further tested G broths with different MSG concentrations to determine whether a better concentration for maximum GABA production existed. Following *L. brevis* RK03 growth in G broths with different MSG concentrations (250–850 mM), 1 × 10^9^ CFU/mL initial inoculum, and pH 4.5 at 30 °C for 96 h, we found that 650 mM MSG yielded the maximum GABA production (Figure 4A). Assessment throughout the growth time course indicated that cells grown in G broth containing 650 mM MSG produced a maximum GABA yield of 62,523 mg/L at 88 h, with slightly lower production (57,591 mg/L) at 96 h (Figure 4B). Thus, G broth containing 650 mM MSG (referred to as GM broth) and growth for 88 h at 30 °C is ideal for GABA production.

### 2.7. Acid, Intestinal, Gastrointestinal Juice Tolerances and Antibiotic Susceptibilities

It is important to know whether potential probiotic bacteria such as *L. brevis* RK03 can tolerate acids and intestinal and gastrointestinal juices. If they are resistant to strong acids in stomach, they can survive in the intestine as live probiotics. Examination of *L. brevis* RK03 viability following acid or juice exposure demonstrated that, even after 3 h at pH 2.0, the *L. brevis* RK03 survival rate approximated 65% with an initial inoculum of around 1 × 10^7^ CFU/mL, whereas it approximated 78% at pH 2.5 and 79% at pH 3 (Table 2). At the highest bile salt concentration tested (0.45%), the cell number did not decrease significantly compared to that in controls after 24 h of treatment. After 3 h, 66.6% of cells remained in gastric juice medium and, after 12 h, approximately 56% survived intestinal juice treatment (Table 2). Thus, over 56% of cells survived at a strongly acidic pH of 2.0 and in the presence of 0.45% bile, or gastric or intestinal juice media. In the antibiotic resistance test, we found that *L. brevis* RK03 is resistant to vancomycin, streptomycin, and spectinomycin but sensitive to ampicillin, tetracycline, chloramphenicol, kanamycin, and erythromycin (Table 3). These data are similar to those of Rushdy and Gomaa [34], who found that *L. brevis* B23 is resistant to vancomycin and some other antibiotics. 

## 3. Discussion

To the best of our knowledge, this is the first study to evaluate a GABA-producing LAB obtained from deep-sea bigeye fish. In this study, thirty-two *Lactobacillus* strains isolated from the fish were evaluated for GABA-producing ability. Of the thirty-two strains, RK03 showed the highest GABA production (15,143 mg/L) in MRS broth containing 550 mM MSG after 96 h of incubation. The effects of growth temperature, incubation time, initial pH, and initial glutamic acid concentration on GABA production by *L. brevis* RK03 were then further investigated. The pH of culture medium changes with incubation time during fermentation, and therefore the initial pH influenced the final biomass and GABA production. We found that the optimal initial pH value was 4.5. The maximum GABA production was observed at 550 mM MSG. In addition, for fermentation, we found that the optimal recipe for the maximum production of GABA (G broth) included 1% glucose; 2.5% yeast extract; 2 ppm each of CaCO_3_, MnSO_4_, and Tween 80; and 10 μM PLP. Using this recipe, we further assessed the growth time course and determined that maximal GABA production (62,523 mg/L) was achieved with 650 mM MSG after 88 h. 

The MRS medium used to grow most *L. brevis* strains is complex and costly. Therefore, it is important to reduce growth medium costs and determine effective medium components for industrial use. For this reason, an inexpensive source of nutrients available on a large scale is of the highest priority. In this study, we defined GM broth components for scaling *L. brevis* RK03 fermentation. GM broth contains 1% glucose; 2.5% yeast extract; 2 ppm each of CaCO_3_, MnSO_4_, and Tween 80; 10 μM PLP; and 650 mM supplemented MSG. Its cost is approximately 35.2-fold cheaper than that of MRS medium, and it increases GABA yield (g/L) by 2.5-fold (Table S2). Moreover, GABA production cost using *L. brevis* RK03 and GM broth is approximately 8.53-fold cheaper than that using *L. brevis* NCL912 (USD/kg) (Table S2).

Li et al. fermented *L. brevis* NCL912 in Erlenmeyer flasks and produced a maximum GABA yield of 35.66 g/L with a conversion rate of 69.12% [14]. Furthermore, Li et al. used the same strain for fed-batch fermentation to produce GABA and obtained a maximal yield of 103 g/L GABA [15]. In this study, MSG was added into fermentation medium at three separate time points for a total of 1395.9 mM MSG, and final GABA production was 1005.81 mM for a conversion rate of 72.05%. Binh et al. found that *L. brevis* K203, isolated from kimchi, produced approximately 44.4 g/L GABA after fermenting in their optimized medium for 72 h, with a conversion rate of around 99.7% based on adding 6% glutamate [13]. Villegas et al. used *L. brevis* CRL1942 to ferment GABA in MRS, adding 270 mM MSG and fermenting for 48 h, and produced 26.3 g/L GABA at a conversion rate of around 90% [16]. Compared to these results, we found that fermenting *L. brevis* RK03 in flasks produced 62.523 g/L GABA after adding 650 mM MSG, for a conversion rate of 93.28%. This suggests that *L. brevis* RK03 has a better conversion rate than did the previous highest-producing strain, *L. brevis* NCL912. Although *L. brevis* K203 had a higher conversion rate than *L. brevis* RK03, the latter strain produces much more GABA.

When LAB enter the human digestive system, where gastric acid and intestinal juices are produced, surviving LAB are absorbed into the intestinal tract, improving the human intestinal bacteria profile and aiding digestion. Once *Lactobacillus* enters the gastrointestinal tract, its survival is dependent on its ability to tolerate acid and bile. Therefore, the ability of potential probiotic bacteria such as *L. brevis* RK03 to tolerate acids as well intestinal and gastrointestinal juices is highly relevant, as probiotic bacteria are consumed in health food products. Previously, Liu et al. found that when *Lactobacillus zeae* LB1 was exposed to a continuous, simulated gastrointestinal acid test for 2 h, cells were reduced to around 2.55 log CFU/mL, whereas when exposed to simulated intestinal fluid for 4 h, cell number was not reduced [35]. Rönkä et al. found that when the *L. brevis* strains GRL1 and GRL62 were inoculated into MRS medium at an initial pH of 2 for 3 h, the number of cells decreased by 5.91 and 2.96 log CFU/mL, respectively [36]. Uroić et al. assessed *L. brevis* D6 growth in a continuous, simulated gastrointestinal acid test for 4 h and observed reductions of 2 log CFU/mL [37]. We found that, even after 3 h, the survival rates of *L. brevis* RK03 at pH 2, 2.5, and 3 were around 65%, 78%, and 79%, respectively, with an initial inoculum of 1 × 10^7^ CFU/mL (Table 2). This means that the cell death rate was <1 log CFU/mL, a large improvement over that observed in other studies.

Although human bile ingredients and concentrations differ from those of bovids, many researchers use bovine bile salts for in vitro tests [38,39]. For example, they were used by Rönkä et al. to assess *L. brevis* GRL1 and GRL62 for 3 h indicated that *L. brevis* GRL1 cell numbers decreased, whereas *L. brevis* GRL62 was not significantly affected [36]. Kimoto et al. tested the fermentation of *L. brevis* KB290 inoculated in MRS and TYGA broths containing 0.3% bovine bile salts and found significantly reduced cell numbers after five days [40]. According to Ramos et al., when *L. plantarum* CH3, *L. plantarum* CH41, *L. fermentum* CH58, *L. brevis* FFC199, *L. plantarum* SAU96, and *L. brevis* SAU105 were cultured in MRS containing 0.3% bovine bile salts for 3 h, cell growth was observed and cell numbers increased [11]. Based on these data, different *Lactobacillus* spp. appear to exhibit different tolerances to bovine bile salts. For *L. brevis* RK03, at the highest bile salt concentration (0.45%), cell number was not significantly lower compared to that of controls after 24 h of treatment. Overall, *L. brevis* RK03 appears more resistant to bovine bile salts than do other *Lactobacillus* strains, suggesting that it exhibits a more robust tolerance to both gastric acid and bile salts. 

Barrett et al. grew *L. brevis* DPC6108 under a feces-based fermentation condition and significantly increased the GABA concentration. This suggests that *L. brevis* DPC6108 might be able to survive in human intestines and produce GABA [41]. In vivo tests in an animal model are necessary to determine whether *L. brevis* could produce GABA or increase GABA production in human intestines. Some *L. brevis* strains can also produce biogenic amines [29,30]. Russo et al. found that *L. brevis* IOEB 9809 produces biogenic amines such as tyramine and putrescine when the culture media contains precursors like tyrosine and agmatine; under mild gastric stress conditions, they also showed that the survival rate of *L. brevis* IOEB 9809 increases [30]. Similar results were found by Romano et al. [29]. When *L. brevis* is cultured under strong acidic conditions (pH 2.0) with medium containing 50 mM ornithine, it produces putrescine to a concentration of up to 31 mM and increasing survival rate too. Therefore, it is important to examine whether *L. brevis* RK03 can produce biogenic amines. In summary, it is necessary to test *L. brevis* RK03 for biogenic amine production and perform whole genome sequencing analysis, and animal model experiments to determine its potential for use as a probiotic in the future.

## 4. Materials and Methods

### 4.1. Isolation and Identification of GABA-Producing LAB

#### 4.1.1. Isolation of GABA-Producing LAB from Fish Intestines

We isolated thirty-two presumptive LAB strains from ocean fish such as *P. macracanthus*, *Chanos chanos*, *Perca fluviatilis*, *Thunnus thynnus*, *Psenopsis anomala*, *Ostreoida Rafinesque*, *Ephippus orbis*, *Ctenopharynodon idellus*, and *Penaeus monodon* from two fish markets located in Nantze and Zuoying districts, Kaohsiung City, Taiwan. The fish were kept on ice (~0 °C) and were then dissected aseptically. The fish intestine contents were homogenized with 0.1% peptone water. 

Homogenized samples (100 μL) were spread onto modified LAMVAB plates (pH 5.0) containing MRS broth with cysteine HCl (0.5 g/L), calcium carbonate (5 g/L), sodium azide (0.2 g/L), and agar (40 g/L), (Difco, Detroit, MI, USA) along with vancomycin hydrochloride (2 µg/mL) [42]. After incubation for 48 h at 30 °C, pure colonies were obtained by streaking on MRS agar. The putative LAB isolates were confirmed by Gram staining, acid production capability tests [43], and catalase and oxidase tests.

#### 4.1.2. Gram Staining, Catalase, Oxidase, and Acid Production Tests

For the Gram staining test, the isolated bacteria were examined using a gram staining kit (Baso Diagnostic, Inc., New Taipei, Taiwan) according to the Collins et al. technique, and observed under a light microscope (Leica DM500, Wetzlar, Germany) at 1000× magnification. For the catalase test, bacterial cells were mixed with 3% H_2_O_2_; bubble formation indicated catalase-positive strains. For the oxidase test, one drop of 1% N,N,N′,N′-tetramethyl-*p*-phenylenediamine dihydrochloride was dropped onto the colonies, with the resulting blue color indicating positive strains. For the acid production test, strains were grown on LAMVAB-A plates. Colonies exhibiting transparent rings represented positive strains. Each experiment was performed in triplicate.

#### 4.1.3. Amplification and Sequencing of 16S rDNA

Chromosomal DNA was isolated from *L. brevis* RK03 cells grown in MRS medium around OD_600_ 1.5 for 1 mL, using a Wizard Genomic DNA Purification kit (Promega, Madison, WI, USA). Approximately 500 ng genomic DNA was used as the DNA template. PCR conditions for 16S rDNA amplification consisted of 30 cycles of 95 °C (1 min), 62 °C (30 s), and 72 °C (1 min), plus one additional cycle with a final 5-min incubation at 72 °C for chain elongation. The primers used were 27F (5′-AGAGTTTGATCMTGGCTCAG-3′) and 1492R (5′-CGGTTACCTTGTTACGACTT-3′). The amplification products were purified using the DNA Clean & Concentrator™-5 kit (Zymol Research, Irvine, CA, USA). The of PCR products (16S rDNA) were then subjected to cycle sequencing reactions conducted by Tri-I Biotec Co., Ltd. (New Taipei, Taiwan) using an ABI PRISM 3730XL sequencer and primers 27F and 1492R with the BigDye Terminator kit (Applied Biosystems, Waltham, MA, USA); the sequences were analyzed using the national center for biotechnology information (NCBI) GenBank BLAST tool.

#### 4.1.4. Preservation and Culture of LAB Isolates

We isolated thirty-two potential LAB strains from ocean fish intestines. These isolates were stored in MRS broth containing 50% sterile glycerol at −80 °C. The isolates were subcultured twice in MRS at 37 °C for 24 h and used as inoculums for the following experiments.

### 4.2. Measurement of GABA Content

#### 4.2.1. Thin Layer Chromatography (TLC) Assay

Isolated strains were grown on MRS broth (Difco TM, BD; San Jose, CA, USA) plates supplemented with 1% MSG (Vedan, Taichung, Taiwan) at pH 5.0 and 37 °C. The isolated strains were incubated in 9 mL MRS broth in Pyrex tubes at 37 °C without shaking for 96 h. The GABA-containing cell culture was centrifuged and filtered with 0.22 μm filters followed by thin layer chromatography (TLC) analysis. We spotted 0.5 µL bacterial culture supernatant (RK02, RK03, RK41, RK45), 0.5 µL GABA solution (1%), and 0.5 µL MRS medium onto TLC plates. The TLC running solvent contained acetic acid, 1-butanol, and distilled water (3:5:2) with 1% ninhydrin. After TLC analysis, the LC plates were dried at 105 °C. The plates were then photographed using Canon EOS 800D. Each experiment was performed in triplicate.

#### 4.2.2. High-Performance Liquid Chromatography (HPLC) Analysis

A 100-μL culture broth filtered through a 0.22-μm filter was mixed with 0.5 mL OPA working solution (1 mL OPA stock: 40 mg O-phthalaldehyde/mL methanol) with 25 mL of 0.1 M borate buffer and 100 μL β-mercaptoethanol, and filtered again. GABA standard was mixed with 0.5 mL OPA working solution and 20 μL was measured using the following conditions. High-performance liquid chromatography (HPLC) mobile phase A: 0.1 M sodium acetate (98%, Showa Chemical., Ltd., Tokyo, Japan) dissolved in 900 mL deionized water and 500 μL trimethylamine (Merck, Kenilworth, NJ, USA) to 1 L final volume with deionized water. pH was adjusted to 6.7 using hydrochloric acid (Nihon Shiyaku Reagent, Tokyo, Japan). HPLC mobile phase B: methanol (HPLC grade, Merck). All mobile phases were passed through a 0.22-μm membrane filter. Pump flow rate was set at 1.0 mL/min; column temperature, 30 °C; sample injection volume, 20 µL; and compound detection: UV detector at 340 nm. GABA content was determined using the Hitachi 1110 pump and Hitachi 1410 detector (Hitachi High-Technologies Corporation, Tokyo, Japan) equipped with an Ascentis^®^ C18 column with 5-μm diameter, 150-mm length, and 4.6-mm internal diameter (Sigma-Aldrich, St. Louis, MO, USA). GABA amounts were calculated by comparing the peak area with the corresponding GABA standard. Peak-heights were measured by using SISC-LAB chromatography software (Scientific Information Service Corporation, New Taipei, Taiwan). To confirm accurate GABA peak detection, generation of standard curves with known GABA concentrations preceded sample analysis. Each experiment was performed in triplicate [44].

### 4.3. Single Parameter Optimization

To find the optimal fermentation parameters to produce maximum GABA yield, single variable optimization was carried out to analyze the influence of four fermentation parameters including initial glutamic acid concentration (0–650 mM), culture temperature (25–45 °C), initial pH (3.5–6.5), and initial inoculums (7–9 log CFU/mL) on GABA production by *L. brevis* RK03. Cells were first grown in MRS medium and tested with these four parameters in 15 mL Pyrex tubes. For initial inocula, different cell concentrations (7–9 log CFU/mL) were grown in 9.9 mL MRS medium supplemented with 550 mM MSG (approximately pH 6.7) at 37 °C for 96 h. For temperature testing, cells at around 9 log CFU/mL were grown in 9.9 mL MRS medium with 550 mM MSG at different temperatures (25–45 °C) for 96 h. For pH tests, cells around 9 log CFU/mL were grown in 9.9 mL MRS medium with 550 mM MSG at different pH (3.5–6.5) at 37 °C for 96 h. For MSG concentrations assessment, cells around 9 log CFU/mL were grown in 9.9 mL MRS medium with different MSG concentrations (0–650 mM) at 37 °C for 96 h. GABA production was determined at different time points for each condition. Each experiment was performed in triplicate.

### 4.4. Culture Recipes for Larger Scale GABA Production

Approximately 10^9^ CFU/mL bacterial cells in 9.9 mL base broth (1% glucose, 1% yeast extract, and 1% MSG) were grown at pH 4.5, 30 °C for 96 h. Different organic carbon sources, nitrogen sources, pyridoxal phosphate (PLP), substances of growth factor and different MSG concentrations were examined.

#### 4.4.1. Organic Carbon Sources

Bacterial cells were grown in base broth as described in Section 2.7, except the carbon sources were replaced by different sugars including glucose, lactose, sucrose, maltose, galactose, brown sugar, glycerol, molasses, golden sugar, dextrin1 (DE 8–10), dextrin2 (DE 10–12), or starch at 1%.

#### 4.4.2. Nitrogen Sources

Bacterial cells were grown in base broth as described in Section 2.7 except the nitrogen sources were replaced by different sugars including ammonium thiocyanate, alumiuium nitrate, ammonium nitrate, ammonium molybodate, ammonium persulfate, urea, diammonium hydrogen phosphate, tryptone, soya peptone, beef extract, peptone, or yeast extract at 1%. To obtain better percentages of selected carbon and nitrogen sources, different concentrations of glucose (0–3%), and yeast extract (0–3.5%) were examined.

#### 4.4.3. Growth Factors

Bacterial cells were grown in broth containing 1% glucose, 2.5% yeast extract, and 1% MSG supplement with different substances at 2 ppm, including calcium carbonate, calcium chloride, chromium chloride, chromium trichloride, cobalt chloride, dipotassium, hydrogenphosphate, ferric ammonium sulfate, ferrous sulfate, magnesium sulfate, manganese chloride, manganese sulfate, potassium carbonate, potassium chloride, potassium iodide, potassium sulfate, sodium acetate, Tween 20, Tween 80, and zinc sulfate. Different combinations including calcium carbonate, manganese sulfate, Tween 80, calcium carbonate, manganese sulfate, calcium carbonate, Tween 80, manganese sulfate, Tween 80, calcium carbonate, manganese sulfate, and Tween 80 were compared regarding their GABA productivity.

#### 4.4.4. Pyridoxal Phosphate (PLP) Compound

*L. brevis* RK03 were grown in 9.9 mL broth containing 1% glucose, 2.5% yeast extract, 2 ppm each calcium carbonate, manganese sulfate, and Tween 80 with different PLP concentrations (0–100 μM) for GABA production, with GABA measured at each time point.

#### 4.4.5. Monosodium Glutamate (MSG) Concentrations

*L. brevis* RK03 was grown in 2 L G broth (1% glucose, 2.5% yeast extract, 2 ppm each calcium carbonate, manganese sulfate, and Tween 80 with 10 μM PLP) in 3 L flasks with different MSG concentrations (0–650 mM) for GABA production, with GABA measured at each time point.

### 4.5. In Vitro Test of Gastric Acidity: Tolerance to Simulated Intestinal Juice and Gastrointestinal Juice

Approximately 1 × 10^9^ colony forming units (CFU)/mL *L. brevis* RK03 was diluted in phosphate buffered saline (0.2 M, pH 7.0). For the acid tolerance test, 1 mL cell culture was added into 9 mL gastric acidic juice (0.32% pepsin and 0.2% NaCl), adjusted to pH 2.0, 2.5, and 3.0 at 37 °C for 0, 0.5, 1, 2, and 3 h, and plated cell numbers determined. For the simulated intestinal juice test, 1 mL cell culture was added into 9 mL simulated intestinal juice (0.1% trypsin and 0.3% bile) at pH 8.0 at 37 °C for 0, 2, 4, 6, 12, and 24 h and plated cell numbers determined [35]. For the simulated gastrointestinal juice test, cells treated as for the acid tolerance test were added into 10 mL simulated gastrointestinal juice for 2, 4, 6, 12, and 24 h and plated cell numbers determined. Each experiment was performed in triplicate [22].

### 4.6. Resistance of L. brevis RK03 to Antibiotics

Antibiotic susceptibilities of *L. brevis* RK03 was examined by agar overlay diffusion method as described by Cebeci and Gürakan [45] with some modification. MRS agar plates were overlaid with 0.7% soft agar with 100 mL of each culture (2 × 10^8^ CFU/mL), grown overnight in MRS broth at 30 °C, and stood for 1 h at 30 °C. Then paper discs were placed and antibiotics were applied onto the discs. After 48 h incubation at 30 °C, the diameters of inhibition zones were measured. The strains were tested for their susceptibilities against ampicillin, chloramphenicol, cycloheximide, erythromycin, neomycin, streptomycin, spectinomycin, tetracycline, rifampicin and vancomycin. Antibiotics were used at the concentration of 1–128 μg per disc.

### 4.7. Statistical Analysis

Experimental data were analyzed using IBM SPSS Statistics 20 (Chicago, IL, USA). One-way analysis of variance (ANOVA) was used to determine the statistical differences between the sample means, with the level of significance set at *p* < 0.05. Multiple comparisons of the means were conducted using the Doucan’s multiple range tests. All data are expressed as mean ± SD.

## 5. Conclusions

In conclusion, using the newly isolated *L. brevis* RK03, GM broth of 1% glucose; 2.5% yeast extract; 2 ppm each of CaCO_3_, MnSO_4_, and Tween 80; 10 μM PLP; and 650 mM MSG comprises the best recipe for GABA production for 88 h at 30 °C. The discovery of *Lactobacillus* strains with the ability to synthesize GABA may offer new opportunities for designing improved GABA- and probiotic bacteria-enriched health-promoting functional foods. Such strains will facilitate the development of functional fermented foods. However, further study is needed to determine the large-scale fermentation conditions for maximum GABA production.

## Figures and Tables

**Table 1:** The effect of γ-aminobutyric acid (GABA) production by *L. brevis* RK3 grown in Man, Rogosa and Sharpe (MRS) medium with or without monosodium glutamate (MSG) at different initial inoculum cells, temperature, and pH values.

Medium	MRS			MRS-M		
Group	pH Value	Viability (log CFU/mL)	GABA (mg/L)	pH Value	Viability (log CFU/mL)	GABA (mg/L)
**Amount of MSG (mM)**						
0	N/A	N/A	N/A	4.27 ± 0.01	8.63 ± 0.01	773.00 ± 62.23 ^f^
200	N/A	N/A	N/A	5.56 ± 0.01	8.14 ± 0.02	8209.88 ± 326.53 ^e^
400	N/A	N/A	N/A	5.57 ± 0.18	7.98 ± 0.01	11,764.66 ± 4.24 ^d^
450	N/A	N/A	N/A	6.20 ± 0.01	7.96 ± 0.05	13,103.67 ± 477.80 ^d^
500	N/A	N/A	N/A	6.72 ± 0.01	8.21 ± 0.17	13,769.12 ± 852.35 ^c^
550	N/A	N/A	N/A	6.71 ± 0.14	8.14 ± 0.11	15,143.22 ± 182.87 ^a^
600	N/A	N/A	N/A	6.70 ± 0.04	8.28 ± 0.07	14,133.06 ± 113.63 ^b^
650	N/A	N/A	N/A	6.75 ± 0.01	7.96 ± 0.02	14,673.63 ± 455.74 ^ab^
**Cell inoculum (log CFU/mL)**						
7	4.41 ± 0.01 ^a^	8.00 ± 0.01 ^b^	711.00 ± 0.00 ^b^	6.85 ± 0.05 ^b^	9.05 ± 0.06 ^b^	9058.00 ± 321.00 ^b^
8	4.41 ± 0.01 ^a^	8.21 ± 0.01 ^a^	739.00 ± 22.63 ^ab^	6.89 ± 0.01 ^b^	9.08 ± 0.01 ^b^	9209.00 ± 313.00 ^b^
9	4.43 ± 0.01 ^a^	8.28 ± 0.04 ^a^	773.00 ± 9.90 ^a^	6.99 ± 0.01 ^a^	9.30 ± 0.03 ^a^	14,443.00 ± 138.00 ^a^
**Temperature (°C)**						
25	4.25 ± 0.21 ^b^	8.40 ± 0.03 ^ab^	922.80 ± 103.81 ^b^	6.94 ± 0.03 ^ab^	9.06 ± 0.00 ^a^	17,669.33 ± 1442.80 ^b^
30	4.64 ± 0.02 ^a^	8.66 ± 0.02 ^a^	1203.62 ± 34.92 ^a^	6.99 ± 0.10 ^ab^	9.06 ± 0.03 ^a^	21,936.27 ± 635.93 ^a^
35	4.31 ± 0.00 ^b^	8.22 ± 0.13 ^ab^	780.61 ± 108.27 ^bc^	6.95 ± 0.07 ^ab^	9.02 ± 0.04 ^a^	16,839.74 ± 306.42 ^bc^
37	4.38 ± 0.03 ^b^	8.25 ± 0.27 ^ab^	702.89 ± 9.24 ^c^	7.05 ± 0.07 ^a^	9.09 ± 0.01 ^a^	14,721.66 ± 1028.51 ^c^
40	4.34 ± 0.06 ^b^	8.16 ± 0.23 ^ab^	341.97 ± 7.20 ^d^	6.86 ± 0.06 ^b^	8.72 ± 0.01 ^b^	10,779.06 ± 1111.58 ^d^
45	4.47 ± 0.05 ^ab^	8.37 ± 0.01 ^ab^	112.51 ± 8.61 ^e^	5.68 ± 0.01 ^c^	7.56 ± 0.02 ^c^	310.86 ± 8.22 ^e^
**Initial pHs**						
3.5	3.33 ± 0.03 ^c^	5.57 ± 0.04 ^c^	140.63 ± 15.88 ^e^	3.19 ± 0.00 ^f^	7.39 ± 0.11 ^c^	226.56 ± 11.82 ^d^
4.0	4.41 ± 0.11 ^b^	8.03 ± 0.05 ^b^	956.71 ± 44.00 ^ab^	6.52 ± 0.01 ^b^	8.93 ± 0.03 ^ab^	24,684.34 ± 562.31 ^a^
4.5	4.58 ± 0.04 ^ab^	8.29 ± 0.01 ^a^	982.97 ± 44.84 ^ab^	6.70 ± 0.05 ^a^	8.90 ± 0.05 ^b^	25,359.36 ± 541.02 ^a^
5.0	4.53 ± 0.04 ^ab^	8.28 ± 0.09 ^a^	895.54 ± 17.09 ^bc^	6.22 ± 0.02 ^c^	8.88 ± 0.05 ^b^	21,155.93 ± 336.47 ^b^
5.5	4.63 ± 0.10 ^a^	8.15 ± 0.06 ^ab^	807.85 ± 46.91 ^d^	6.18 ± 0.01 ^cd^	8.98 ± 0.02 ^ab^	19,883.72 ± 109.77 ^b^
6.0	4.64 ± 0.06 ^a^	8.13 ± 0.19 ^ab^	818.32 ± 33.38 ^cd^	6.11 ± 0.03 ^de^	9.03 ± 0.08 ^ab^	19,411.73 ± 687.76 ^b^
6.5	4.61 ± 0.06 ^a^	8.25 ± 0.10 ^ab^	765.96 ± 29.60 ^d^	6.11 ± 0.07 ^e^	9.08 ± 0.07 ^a^	11,473.79 ± 709.65 ^c^

MRS: MRS broth without MSG; MRS-M: MRS broth with 550 mM MSG; N/A: Not Available. Data are expressed as mean ± SD from triplicate experiments. Different letters in the column of each group are significantly different (*p* < 0.05).

**Table 2:** Survival of *L. brevis* RK03 exposed to acid for 3 h, bile salts for 24 h, simulated gastrointestinal (GI) juice for 3 h, and intestinal juice for 12 h.

**pH**	**Population Changes Compared to Initial Count/Acid Tolerance Time (log CFU/mL)**							
	**0 h**	**0.5 h**		**1 h**	**2 h**	**3 h**	**Survival Rate (%)**	
2	7.35 ± 0.01 ^bA^	7.06 ± 0.05 ^aAB^		6.47 ± 0.66 ^aBC^	5.78 ± 0.19 ^bC^	4.78 ± 0.01 ^dD^	65	
2.5	7.34 ± 0.02 ^bA^	7.10 ± 0.07 ^aB^		7.04 ± 0.01 ^aB^	5.96 ± 0.10 ^bC^	5.69 ± 0.04 ^cD^	78	
3	7.79 ± 0.00 ^aA^	7.11 ± 0.03 ^aB^		6.97 ± 0.03 ^aC^	6.82 ± 0.00 ^aD^	6.12 ± 0.06 ^bE^	79	
7	7.74 ± 0.05 ^aA^	7.11 ± 0.02 ^aB^		7.11 ± 0.06 ^aB^	7.11 ± 0.06 ^aB^	6.92 ± 0.01 ^aC^	89	
**Bile Salt (%)**	**Population Changes Compared to Initial Count/Bile Salt Tolerance Time (log CFU/mL)**							
	**0 h**	**2 h**	**4 h**	**6 h**	**12 h**	**24 h**	**Survival Rate (%)**	
0	7.82 ± 0.05 ^aA^	7.17 ± 0.13 ^aB^	6.94 ± 0.03 ^aBC^	7.04 ± 0.13 ^aBC^	7.02 ± 0.03 ^aBC^	7.02 ± 0.03 ^aC^	90	
0.15	7.82 ± 0.05 ^aA^	7.08 ± 0.05 ^abB^	7.14 ± 0.05 ^aB^	7.09 ± 0.09 ^aB^	7.08 ± 0.05 ^aB^	7.10 ± 0.28 ^aB^	91	
0.3	7.82 ± 0.05 ^aA^	6.89 ± 0.05 ^bB^	7.21 ± 0.28 ^aB^	7.10 ± 0.04 ^aB^	7.04 ± 0.02 ^aB^	7.04 ± 0.18 ^aB^	90	
0.45	7.82 ± 0.05 ^aA^	7.08 ± 0.09 ^abB^	7.04 ± 0.07 ^aCD^	7.05 ± 0.10 ^aCD^	6.85 ± 0.13 ^aCD^	6.89 ± 0.04 ^aD^	88	
**Cell Survival Ability**	**Gastric Juice at Treated Time**				**Intestinal Juice at Treated Time**			
	**0 h**	**1 h**	**2 h**	**3 h**	**2 h**	**4 h**	**6 h**	**12 h**
Survival count (log CFU/mL)	7.34 ± 0.02 ^A^	6.15 ± 0.01 ^B^	5.71 ± 0.25 ^C^	4.89 ± 0.00 ^D^	4.70 ± 0.06 ^E^	4.27 ± 0.03 ^F^	4.17 ± 0.11 ^F^	4.12 ± 0.18 ^G^
Survival rate (%)	100.0	83.8	77.8	66.6	64.00	58.00	56.80	56.10

Survival rate (%) = (Viable counts (log CFU/mL) for different hours/Viable counts (log CFU/mL) at 0 h) × 100. ^a–^^d^: Value with different letters in the same column differ significantly (*p* ≤ 0.05). Data are expressed as mean ± SD from triplicate experiments. Different letters in the column (^a–^^d^) and row (^A–^^G^) of each group are significantly different (*p* < 0.05).

**Table 3:** Antibiotic susceptibilities of *L. brevis* RK03.

Strain	Am	Cm	Em	Kan	Sm	Sp	Tc	Vm
*L. brevis* RK03	S(2.0)	S(32.0)	S(16.0)	S(128.0)	R	R	S(32.0)	R

S, susceptible; R, resistant. Values in the parentheses are the minimal inhibitory amount (μg). Am, ampicillin; Cm, chloramphenicol; Em, erythromycin; Kan, kanamycin; Sm, streptomycin; Sp, spectinomycin; Tc, tetracycline; Vm, vancomycin.

## Supplementary Materials

Supplementary materials can be found at www.mdpi.com/1422-0067/19/1/143/s1.
