# Supplementary material for: Characterization of a Potential Probiotic Lactobacillus brevis RK03 and Efficient Production of γ-Aminobutyric Acid in Batch Fermentation
Source: Int J Mol Sci. 2018 Jan 4;19(1):143. doi: 10.3390/ijms19010143 (PMC5796092; doi:10.3390/ijms19010143)
Supplement: Supplementary file 1 [file ijms-19-00143-s001.pdf]

Supplementary Table S1. Morphological and biochemical characteristics of LABs from various fish intestines.

Strains No.	source	Gram stain	Acid production	Catalase test	Oxidase test	GABA (mg/L)
RK01	1[a]	+	+	-	-	223
RK02	1	+	+	-	-	785
RK03	1	+	+	-	-	1,024
RK05	2	+	+	-	-	106
RK08	2	+	+	-	-	154
RK12	2	+	+	-	-	123
RK13	2[b]	+	+	-	-	138
RK14	2	+	+	-	-	237
RK15	3	+	+	-	-	141
RK17	3	+	+	-	-	469
RK18	3	+	+	-	-	543
RK19	4[a]	+	+	-	-	639
RK20	4	+	+	-	-	99
RK22	4	+	+	-	-	309
RK24	4	+	+	-	-	473
RK26	5	+	+	-	-	76
RK27	5	+	+	-	-	152
RK29	5	+	+	-	-	629
RK30	5	+	+	-	-	529
RK35	6	+	+	-	-	218
RK36	6	+	+	-	-	282
RK37	7[b]	+	+	-	-	289
RK38	7	+	+	-	-	237
RK41	8[a]	+	+	-	-	730
RK42	8	+	+	-	-	578
RK43	8	+	+	-	-	416
RK45	8	+	+	-	-	813
RK46	9	+	+	-	-	252
RK47	9	+	+	-	-	239
RK48	9	+	+	-	-	223
RK49	9	+	+	-	-	165
RK50	9	+	+	-	-	216

1 : *Priacanthus macracanthus*; 2 : *Chanos chanos* ; 3: *Perca fluviatilis* ; 4: *Thunnus thynnus* ; 5: *Psenopsis anomala* ; 6: *Ostreoida Rafinesque* ; 7: *Ephippus orbis* ; 8: *Ctenopharynodon idellus* ; 9: *Penaeus monodon* ;. [a]: Kaohsiung Nanzih ; [b]: Kaohsiung Zuoying.

Supplementary Table S2. The comparison of cost of the fermentation media, MRS medium and GM broth.

	g/Bottle	Unit price $USD	$USD/g	Requirements g/L	$USD/L	GABA g/L	GABA $USD/kg
**MRS-MSG**							
MRS	500	120	0.240	55.00	13.20		
MSG	500	2.65	0.005	102.92	0.55		
Total					13.75	25.36	542.2
**GM broth**							
Glucose	500	1.43	0.003	10.00	0.030		
Yeast extract	4,000	50.43	0.013	25.00	0.325		
MSG	500	2.65	0.005	121.63	0.608		
$CaCO_3$	500	33.18	0.066	0.002	0.0001		
$MnSO_4$	500	43.13	0.086	0.002	0.0002		
Tween 80	1,000	47.78	0.048	0.002	0.0001		
PLP	1	23.56	23.56	0.003	0.0001		
Total					0.96	62.52	15.4
***Lactobacillus brevis* NCL912 (Li *et al.*, 2010b)**							
Glucose	500	1.43	0.003	50.00	0.150		
Soya peptone	100	49.00	0.490	25.00	12.250		
MSG	500	2.65	0.005	243.26	1.216		
$MnSO_4$	500	43.13	0.086	0.01	0.001		
Tween 80	1,000	47.78	0.048	0.01	0.001		
Total					13.62	103.72	131.3
